# Comparative structure-function features of Hsp70s of *Plasmodium falciparum* and human origins

**DOI:** 10.1007/s12551-019-00563-w

**Published:** 2019-07-06

**Authors:** Graham Chakafana, Tawanda Zininga, Addmore Shonhai

**Affiliations:** Department of Biochemistry, University of Venda, Private Bags X5050, Thohoyandou, 0950 South Africa

**Keywords:** Hsp70, Molecular chaperone, Functional specificity, Signature motif, *Plasmodium falciparum*, Human

## Abstract

The heat shock protein 70 (Hsp70) family of molecular chaperones are crucial for the survival and pathogenicity of the main agent of malaria, *Plasmodium falciparum*. Hsp70 is central to cellular proteostasis and some of its isoforms are essential for survival of the malaria parasite. In addition, they are also implicated in the development of antimalarial drug resistance. For these reasons, they are thought to be potential drug targets, especially in antimalarial combination therapies. However, their high sequence conservation across species presents a hurdle with respect to their selective targeting. The human genome encodes 17 Hsp70 isoforms while *P. falciparum* encodes for only 6. The structural architecture of Hsp70s is typically characterized by a highly conserved N-terminal nucleotide-binding domain (NBD) and a less conserved C-terminal substrate-binding domain (SBD). The two domains are connected by a highly conserved linker. In spite of their fairly high sequence conservation, Hsp70s from various species possess unique signature motifs that appear to uniquely influence their function. In addition, their cooperation with co-chaperones further regulates their functional specificity. In the current review, bioinformatics tools were used to identify conserved and unique signature motifs in Hsp70s of *P. falciparum* versus their human counterparts. We discuss the common and distinctive structure-function features of these proteins. This information is important towards elucidating the prospects of selective targeting of parasite heat shock proteins as part of antimalarial design efforts.

## General structure-function features of Hsp70

**Fig. 1:**
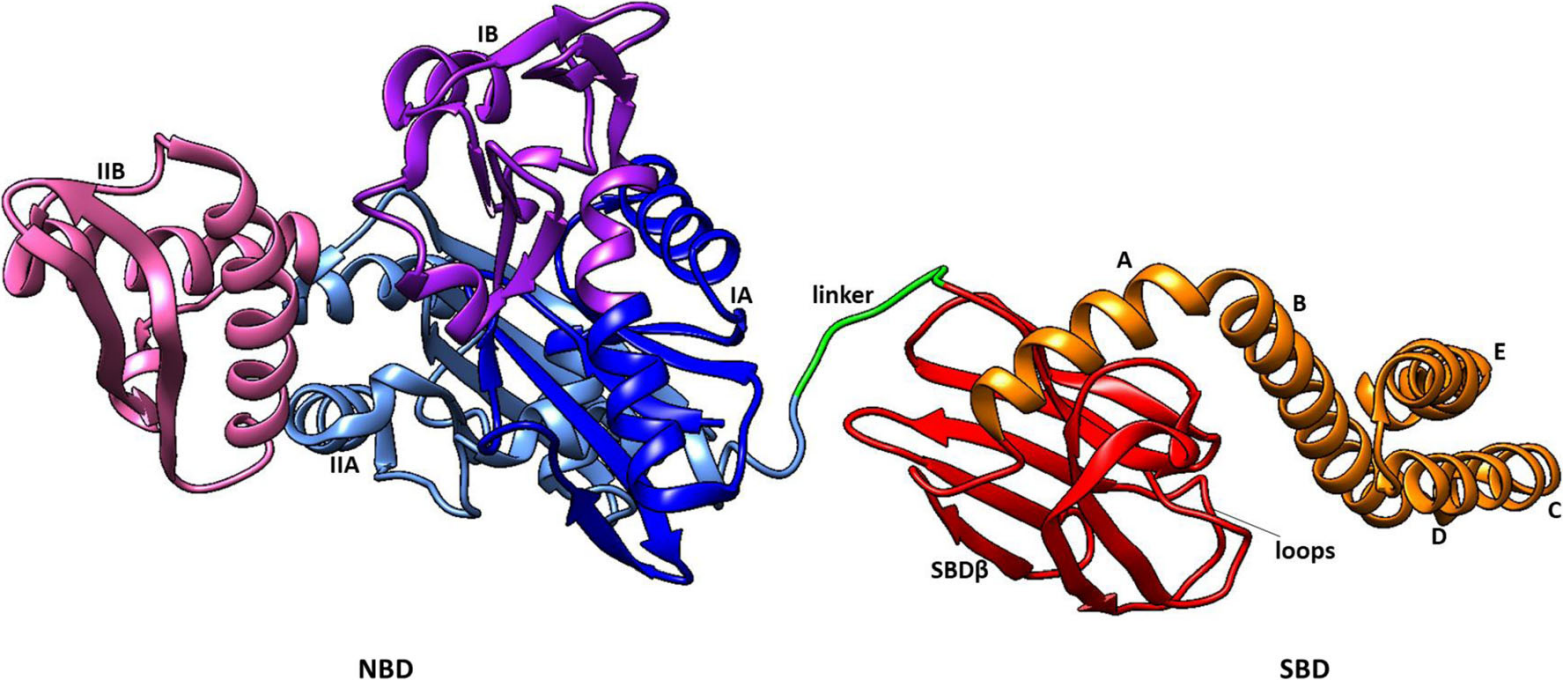
General structure of Hsp70s. The NBD is comprised of lobes IA (blue), IIA (blue), IB (purple) and IIB (pink). The SBD is constituted by SBD-β (red) and SBD-α subunits (orange) of which the latter is subdivided into helices A–E. The NBD and SBD are adjoined by a highly conserved linker (green)

The Hsp70 members are central mediators of cellular proteostasis. They facilitate de-novo protein folding of nascent polypeptides, protein translocation and are implicated in signal transduction (Mayer 2010). Hsp70s thus interact with proteins at virtually every stage of their life cycles, from primary folding through to degradation. Hsp70s are strongly upregulated by physiological stress. Their cooperation with other Hsp members such as Hsp110, Hsp100, Hsp90, Hsp60, Hsp40 and the small Hsps enhances their functional versatility (Mogk et al. 2015). Hsp70s possess a highly conserved N-terminal nucleotide-binding domain (NBD) and a more varied C-terminal substrate-binding domain (SBD), adjoined by a linker (Fig. 1; Mayer 2010). The SBD of Hsp70 is reportedly functionally promiscuous as it allows the chaperone to bind to short degenerate motifs within peptide substrates (Rosenzweig et al. 2017). This technically provides Hsp70 with the capability to bind to virtually all proteins.

Generally, Hsp70s of prokaryotic and mammalian family members share at least 50% identity, making Hsp70 one of the most conserved proteins (Yu et al. 2015). Hsp70s are generally ubiquitous, accounting for approximately 1–2% of the cellular proteome (Dhamad et al. 2016). Several organisms express multiple Hsp70s. For instance, while humans express a complement of 17 Hsp70s, the major malaria parasite, *Plasmodium falciparum*, expresses 6 Hsp70s (Kampinga et al. 2009). The various Hsp70 isoforms localize to different subcellular compartments. Hsp70s particularly play a crucial role in the survival of *P. falciparum*, since in its development, the parasite cycles between a poikilothermic mosquito vector and a homoeothermic human host. The development of malaria fever at the blood stage is closely linked to the modification of the infected human red blood cell (RBC), making it cytoadherent, thus leading to clinical complications of malaria. Not only does physiological stress upregulate general expression of parasite Hsps, but some of these are exported to the infected RBC (Pryzborski et al. 2015). Amongst the exported proteins are several Hsp40 members and *P. falciparum* Hsp70-x (PfHsp70-x; Külzer et al. 2012). Hsp40 co-chaperones serve as substrate scanners for Hsp70 and also stimulate the otherwise, functionally rate limiting ATPase activity of the latter (Botha et al. 2011). Consequently, though not essential, PfHsp70-x along with some exported parasite Hsp40s are implicated in augmenting pathogenicity of the malaria parasite (Cobb et al. 2017). Because of their implications in parasite survival, antimalarial drug resistance and pathogenicity, Hsps of *P. falciparum* are prospective drug targets (Shonhai 2010). Here, we conducted systematic analyses on the structure-function features of Hsp70s from *P. falciparum* and human systems towards identifying unique features towards selective targeting of parasite Hsp70s towards the development of novel antimalarials.

## Subcellular localization and functional features of *P. falciparum* Hsp70s and their human homologues

**Fig. 2:**
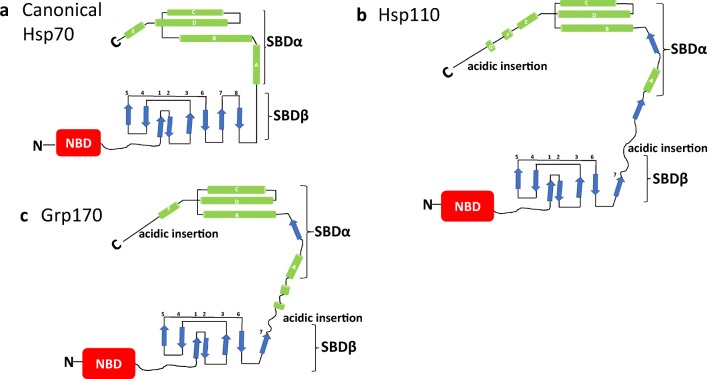
Domain architecture of Hsp70 superfamily. The schematic represents canonical and non-canonical Hsp70 structures. The SBD_β_ is composed of eight sheets while SBDα is constituted by helices A–E (**a**). Hsp110s, of which PfHsp70-z is a member possess insertions in SBDα and towards the lid segment (**b**). Grp170 possesses more acidic insertions in SBDα and another leading to the C-terminus (**c**). The figure was adapted from Oh et al. (1999)

*P. falciparum* expresses 6 Hsp70 members which are located in various subcellular compartments: PfHsp70-1 and PfHsp70-z (cytosol) PfHsp70-2 and PfHsp70-y (ER), PfHsp70-3 (mitochondrium) and PfHsp70-x which occurs in the parasitophorous vacuole and is also exported to the infected RBC (Table 1; Külzer et al. 2012; Shonhai 2014). Hsp70s are classified into two main groups: canonical Hsp70s which structurally resemble *E. coli* Hsp70 (DnaK), while the larger in size, Hsp110/glucose regulated protein 170 (Grp170) members (Dragovic et al. 2006), constitute the non-canonical Hsp70s. The latter possess chaperone function which is limited largely to suppression of protein aggregation, while the former are efficient protein folders. Apart from their role as chaperones, Hsp110s are thought to serve as nucleotide exchange factors (NEFs) of their canonical Hsp70 counterparts (Dragovic et al. 2006). Structurally, Hsp110s are marked by extended acidic insertions located within their SDB-β and the SBD-α subunits and they possess linker segments that are distinct from those present in canonical Hsp70s (Fig. 2; Oh et al. 1999). PfHsp70-1 (cytosol/nucleus), PfHsp702 (ER) and PfHsp70–3 (mitochondrium) constitute the canonical Hsp70 isoforms of *P. falciparum*. PfHsp70-z (cytosol) and PfHsp70-y (ER) belong to the Hsp110 and Grp170 families, respectively. On the other hand, humans express 13 Hsp70s, 3 Hsp110s and 1 Grp170. The Hsp70 isoforms carry out specialized protein folding functions within their various subcellular locations (Tables 1 and 2, Kampinga et al. 2009; Shonhai 2014).

**Table 1:** Characteristic features of *P. falciparum* Hsp70s

PfHsp70 (PlasmoDB accession number)	Size (kDa)	Localization	Stress Inducible (yes/no)	Cellular functions	References
PfHsp70-1 (PF3D7_0818900)	74	Nucleus and cytosol	Yes	Protein folding/translocation/aggregation suppression	Shonhai et al. 2007 Shonhai et al. 2008
PfHsp70-z (PF3D7_0708800)	100	Cytosol	Yes	Predicted NEF of PfHsp70-1; aggregation suppression	Muralidharan et al. 2012 Zininga et al. 2016
PfHsp70-2 (PF3D7_0917900)	73	E.R	Yes	Protein import and folding in the ER, retrograde translocation of proteins for degradation	Shonhai et al. 2007 Chen et al. 2018
PfHsp70-y (PF3D7_1344200)	108	E.R	ND	Thought to be NEF for PfHsp70-2	Shonhai et al. 2007 Njunge et al. 2013
PfHsp70-3 (PF3D7_1134000)	73	Mitochondrium	ND	Protein translocation into the mitochondrium	Shonhai et al. 2007
PfHsp70-x (PF3D7_0831700)	76	P.V and exported to parasite infected RBC	Yes	Protein export and subsequent protein folding of exported proteins in the infected RBC	Charnaud et al. 2017 Cobb et al. 2017

ND not determined

**Table 2:** Subcellular localization of human Hsp70s

Human Hsp70 (accession number)	Size (kDa)	Localization	Stress inducible (yes/no)	Cellular functions	References
1. HspA1A/Hsp70-1 (P0DMV8)	70	Cytosol, nucleus, cell membrane, extracellular exosomes	Yes	Protein folding, translocation and facilitating degradation of misfolded proteins	Khalouei et al. 2014 Radons 2016
2. HspA1B/Hsp70-2 (P0DMV9)	70	Cytosol, nucleus, extracellular exosomes	Yes	Protein folding, translocation and facilitating degradation of misfolded proteins	Radons 2016
3. HspA1L/Hsp70-1L (P34931)	70	Cytosol, nucleus	No	Aggregation suppression, protein folding, facilitates spermatogenesis	Zhu et al. 1997
4. HspA2 (P54652)	70	Cytosol, nucleus, cell membrane, extracellular exosomes	Yes	Protein folding and aggregation suppression	Pigłowski et al. 2007 Redgrove et al. 2012
5. HspA8/Hsc70 (P11142)	71	Cytosol, nucleus, cell membrane, extracellular exosomes	No	Protein folding, translocation, repression of transcriptional activation	Dworniczak and Mirault 1987
6. HspA14 (Q0VDF9)	55	Cytosol, cell membrane	Yes	Component of the ribosome-associated complex (RAC), which is involved in folding or maintaining nascent polypeptides in a folding-competent state	Wu et al. 2011
7. HspH1 (Q92598)	97	Cytosol, nucleus, endocytic vesicle	Yes	Apoptosis suppression, aggregation suppression, NEF	Zappasodi et al. 2015
8. HspA4L/HspH3 (O95757)	95	Cytosol, nucleus	Yes	Elicits humoral immune responses in leukaemia patients	Takahashi et al. 2007
9. HspA4/HspH2	95	Cytosol, extracellular exosome	N.D	Implicated in spermatogenesis	Held et al. 2011
10. HspA5/BiP (P11021)	72	E.R, extracellular exosomes	No	Protein import and folding in the ER, retrograde translocation of proteins for degradation	Yu et al. 2015
11. HspA13 (P48723)	52	E.R, extracellular exosomes, microsomes	No	Exhibits peptide-independent ATPase activity	Otterson et al. 1994
12. HspH4/Grp170 (Q9Y4L1)	111	E.R	Yes	Aggregation suppression, NEF	Behnke et al. 2015
13. HSPA9/Grp75 (P38646)	74	Mitochondria, nucleus	No	Protein translocation into the mitochondria	Mizzen et al. 1989
14. HspA7 (P48741)	40	Blood microparticles and extracellular exosomes	Yes	N.D	Brocchieri et al. 2008
15. HspA12B (B7ZLP2)	76	Endothelial cells, intracellular, blood plasma	No	N.D	Han et al. 2003 Radons 2016
16.HspA6	71	Cytosol, extracellular exosomes	Yes	Protein folding	Khalouei et al. 2014 Leung et al. 1990
17.HspA12A	75	Intracellular and extracellular exosomes	No	ND	Han et al. 2003 Radons 2016

ND not determined

## Structural features of *P. falciparum* Hsp70s and their human homologues

Multiple sequence alignments between Hsp70s of *P. falciparum* and their homologues of human origin show high conservation which is enhanced for isoforms that occur in respective subcellular locations (Table 3). For example, the cytosolic homologues (PfHsp70-1 and human HspA1A), as well as the E. R homologues (PfHsp70-2 and human HspA5/Bip) showed high identity scores of 72.23 and 65.18%, respectively. PfHsp70-x which is exported to the RBC by the parasite is highly identical to cytosol localized homologues (PfHsp70-1 and human HspA1A). Mabate et al. (2018) established that PfHsp70-x preferentially binds substrates with asparagine repeat rich regions. Nearly 10% of the malaria parasite proteome is characterized by prion-like repeats and at least 30% of the proteome is characterized by glumatate/asparagine repeat segments (Pallarès et al. 2018). Thus, the substrate preference of PfHsp70-x suggests that it may bind and refold malarial proteins that are exported to the parasite-infected RBC. This may be important for the parasite as nearly 500 of its proteins are thought to be exported to the RBC (Hiller et al. 2004). However, PfHsp70-x was shown to be not essential in *P. falciparum* lab strain (Charnaud et al. 2017). However, its export to the RBC was shown to correlate with the early stages of parasite development characterized by rapid remodelling of the RBC (Cobb et al. 2017).

**Table. 3:** Comparative identities of plasmodial and select human Hsp70s

	PfHsp70-y	PfHsp70-z	PfHsp70-3	HspA9	Hsc70	HspA1A	PfHsp70-1	PfHsp70-x	PfHsp70-2	HspA5/BiP
PfHsp70-y	–	23.28	21.04	21.34	24.17	24.36	21.22	22.55	24.88	24.68
PfHsp70-z	23.28	–	21.22	21.27	24.84	24.41	22.71	25.16	21.72	24.31
PfHsp70-3	21.04	21.22	–	62.22	49.92	49.92	47.44	49.06	50.00	51.21
HspA9	21.34	21.27	62.22	–	51.14	50.65	47.86	48.69	47.69	49.84
Hsc70	24.17	24.84	49.92	51.14	–	85.80	71.83	71.00	62.04	65.43
HspA1A	24.36	24.41	49.92	50.65	85.80	–	72.23	71.47	59.16	63.81
PfHsp70-1	21.22	22.71	47.44	47.86	71.83	72.23	–	77.61	55.89	62.32
PfHsp70-x	22.55	25.16	49.06	48.69	71.00	71.47	77.61	–	57.08	62.46
PfHsp70-2	24.88	21.72	50.00	47.69	62.04	59.16	55.89	57.08	–	65.18
HspA5/BiP	24.68	24.31	51.21	49.84	65.43	63.81	62.32	62.46	65.18	–

The percentage identities of the select Hsps were generated after multiple sequence alignments (MSA) of the amino acid sequences of the proteins retrieved from (www.uniprot.org) for human and (www.plasmoDB.org) for *P. falciparum* proteins, respectively. The MSA were conducted using the BioEdit pairwise tool (Hall et al. 2005)

### Multiple sequence alignment of *P. falciparum* Hsp70s and their human homologues

**Fig. 3:**
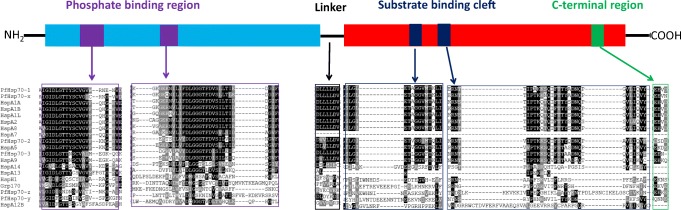
Comparison of domains of plasmodial and human Hsp70s. PfHsp70-y and PfHsp70-z exhibit unique features in the following segments: phosphate binding region, linker, substrate binding clefts and C-terminal regions. There is however higher sequence conservation within the canonical Hsp70s with HspA7, HspA13 and HspA14 exhibiting the greatest variation

Generally, Hsp70s exhibit higher sequence conservation in the NBDs as compared to the SBDs (Fig. 3). The phosphate-binding region of *P. falciparum* Hsp70s is more conserved across canonical Hsp70s than it is within the non-canonical Hsp70 family (Hsp110). This could account for the reported differences in the affinities for nucleotide binding and ATP hydrolysis rates between canonical and non-canonical Hsp70s (Zininga et al. 2016). Residues such as Asp10 and Glu175 in subdomain IA, Lys71 in subdomain IB and Asp199 and Thr204 in subdomain IIA, respectively, are highly conserved and act as interaction sites for ADP (Arakawa et al. 2011). The SBDs of non-canonical Hsp70s are less conserved in comparison to those of their canonical counterparts. As such, the two Hsp70 subclasses are reported to exhibit varied substrate preferences (Zininga et al. 2016). Hsp110s and Grp170s are thought to preferentially bind bulky substrates possessing aromatic residues with a higher affinity than their canonical counterparts (Polier et al. 2008, 2010). We further observed that HspA13 possesses a less conserved substrate-binding cleft (SBC) while HspA14 lacks a typical SBC (Fig. 3). HspA13 is an E.R- and microsome-localized protein which could potentially augment the activity of its E.R counterpart HspA5/BiP. Notably, the human Hsp70 complement is not only more expanded in number but is more structurally diverse than that of the parasite, suggesting that the human protein folding system is more versatile than that of the parasite.

Non-canonical Hsp70s show species-specific variation between the human and *P. falciparum* homologues. PfHsp70-y and PfHsp70-z exhibit unique features in several regions: the phosphate-binding region, linker, substrate-binding clefts and C-terminal segments. The variations in the phosphate-binding regions of canonical versus non-canonical Hsp70s which transcend across the human and *P. falciparum* systems may account for the unique nucleotide binding and hydrolysis rates reported in Hsp70 homologues of these species (Table 4). For example, the linker of human Hsp110 is composed of the residues ^392^EFSVTD^396^ as compared to residues ^422^EYECVE^427^ present in PfHsp70-z (Zininga et al. 2016). Unlike its cytosolic canonical Hsp70 homologues, human HspA14 harbours a less conserved linker (^387^DSLMIEC^392^). Since the Hsp70 linker is implicated in allosteric communication (English et al. 2017), human HspA14 may thus coordinate allostery in a unique fashion.

**Table 4:** Comparative ATP hydrolysis between cytosolic *P. falciparum* Hsp70s and other Hsp70s

Protein	ATP affinity *K*_D_ value (nM)	ATP hydrolysis *K*m value (μM)	Reference
PfHsp70–1	3.48e-6^(a)^	428^(a)^	Zininga et al. 2016^(a)^
PfHsp70-x	1.10e-7^(b)^	393^(c)^	Mabate et al. 2018^(b)^; Cockburn et al. 2014^(c)^
PfHsp70-z	2.50e-5^(a)^	283^(a)^	Zininga et al. 2016^(a)^
Human HspA1A	ND	85^(d)^	Bimston et al. 1998^(d)^
*E. coli* DnaK	4.13e-7^(f)^	20^(e)^	Liberek et al. 1991^(e)^, Lebepe 2019^(f)^

Note: the letters in superscripts depict the various citations of authors that are responsible for the corresponding work reported in the table

*ND* not determined

### Comparative structural features of NBDs of Hsp70s

**Fig. 4:**
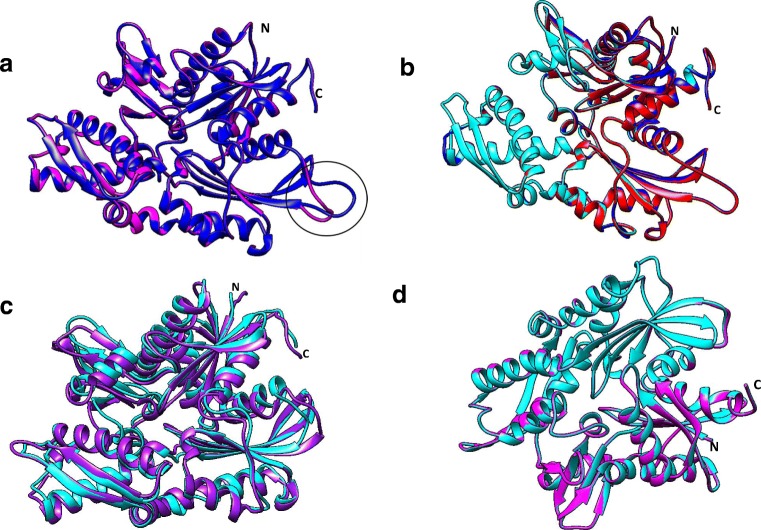
Structural comparison of Hsp70 NBDs. Superimposed images of NBDs of cytosolic Hsp110s: PfHsp70-z (blue), human Hsp110 (magenta) (**a**). Structural variation within the loops in Lobe IIA is encircled. Superimposition of NBDs of cytosolic, canonical Hsp70s shows high structural conservation: PfHsp70-1 (red), HspA1A (blue) and Hsc70 (cyan) (**b**). The ER Grp170, PfHsp70-y (cyan) versus human Grp70 (purple) NBDs show structural variations (**c**). Mitochondrial Hsp70s: PfHsp70-3 (cyan) and human Grp75 (magenta) exhibit high structural conservation in their NBDs (**d**). PfHsp70-2 (cyan) shows minor structural differences from its human ER homologue HspA5 (purple) (**e**). PfHsp70-x (blue) showed greater structural similarity with human HspA1A (magenta) compared to human HspA7 (cyan) (**f**). The variation in the loop segments of lobe IIA is encircled. The three-dimensional models were generated using PHYRE2 (Kelley et al. 2015) and models were visualized using Chimera vs1.1 (Pettersen et al. 2004), and were analysed using Matchmaker plugin to generate images

The NBD of Hsp70 is characterized by an ‘actin-like’ fold composed of 4 subdomains, namely IA, IB, IIA and IIB which form two lobes (lobe I and lobe II) (Fig. 1). These in turn form a hydrophobic nucleotide-binding cleft. Notably, canonical Hsp70s generally exhibited the highest predicted structural conservation in NBDs across species in comparison to the Grp170 group which was the most structurally diverse (Fig. 4). However, NBDs of the Hsp110 protein showed high conservation in both humans and *P. falciparum* with minor variations in a loop connecting the sheet and helical segments in lobe IIA (Fig. 4).

**Fig. 5:**
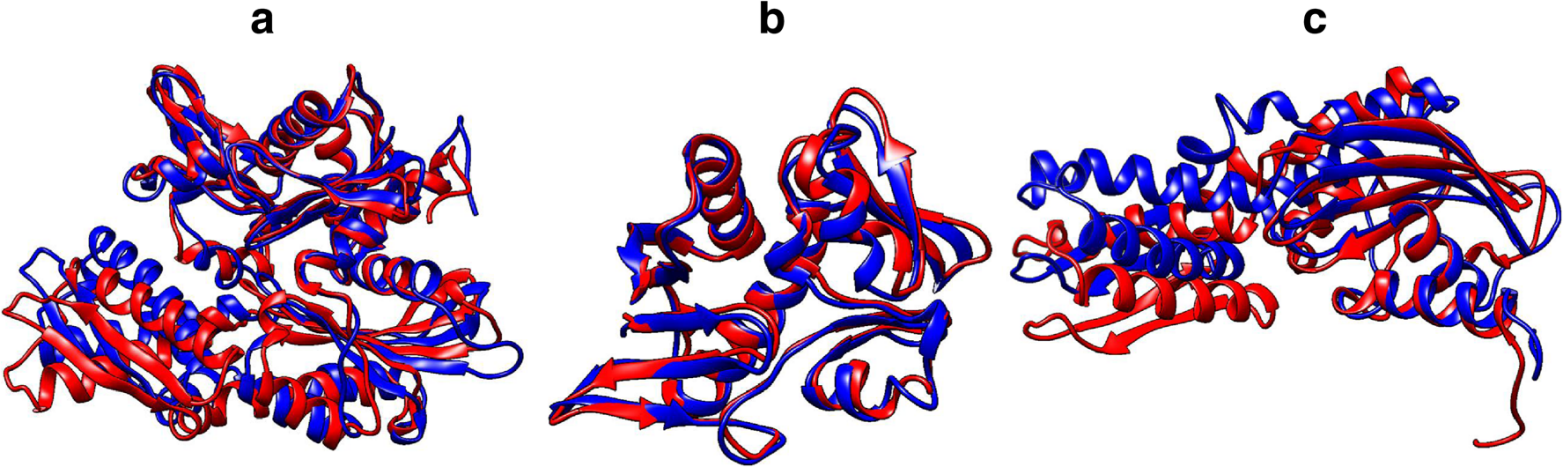
Comparative structural analyses of lobes I and II in NBDs of canonical versus non-canonical Hsp70s. The NBDs of PfHsp70-1 (red) and PfHsp70-z (blue) were superimposed (**a**); (**b**). Lobe I of PfHsp70-1 (red) and PfHsp70-z (blue) exhibits minor structural variations. (**c**). There is more pronounced variation in lobe II of the NBD of PfHsp70-1 and PfHsp70-z. Images were generated using Matchmaker plugin on Chimera 1.11 (Pettersen et al. 2004)

Comparative analysis of the NBDs of the two cytosolic *P. falciparum* Hsp70s (PfHsp70-1 and PfHsp70-z) showed some degree of divergence between these respective canonical and non-canonical Hsp70 family representatives (Fig. 5). While lobe I showed high structural conservation, there was pronounced variation in lobe II of PfHsp70-1 and PfHsp70-z. This predicted structural difference could account for the varied functional features of the two proteins. For example, Zininga et al. (2016) reported that PfHsp70-z possesses nucleotide independent chaperone activity, in contrast to PfHsp70-1 whose activity is regulated by ATP. In addition, PfHsp70-1 has been shown to exhibit higher nucleotide-binding affinity and ATP hydrolysis rates compared to PfHsp70-z (Zininga et al. 2016). Furthermore, *P. falciparum* Hsp70s have also been shown to possess much higher ATPase activity as compared to their human counterparts (Table 2).

### Structural comparisons of SBDs of *P. falciparum* Hsp70s and their human homologues

**Fig. 6:**
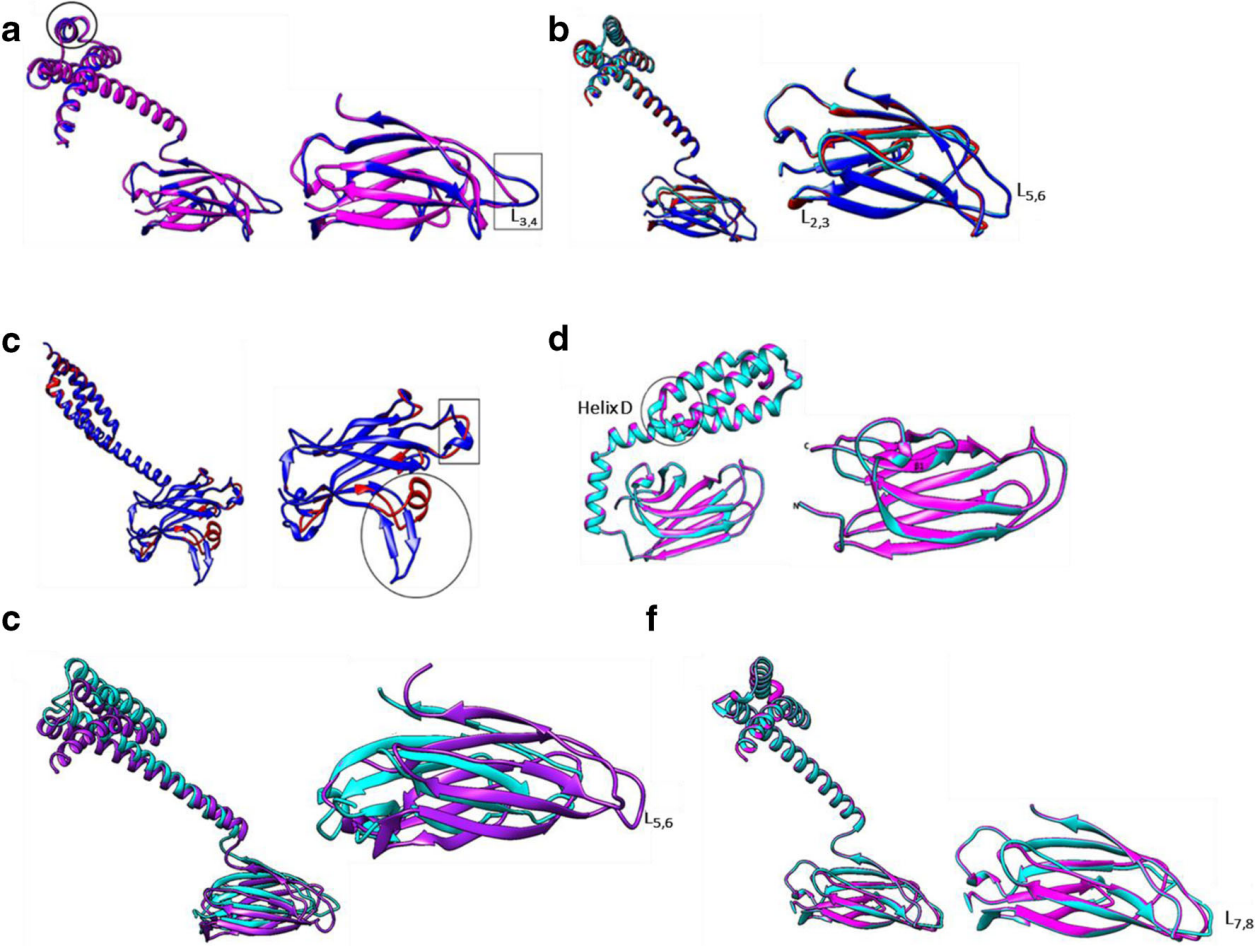
Structural comparison of Hsp70 SBDs The Hsp70. SBDs of cytosolic Hsp110s: PfHsp70-z (blue) versus human HspH1 (magenta) (**a**). **b** SBDs of cytosolic canonical Hsp70s are more conserved: PfHsp70-1 (red), HspA1A (blue) and Hsc70 (cyan). **c** The SBDs of E. R Grp170s: PfHsp70-y (red) versus human Grp170 (blue) show structural uniqueness in SBDβ. **d** Mitochondrial Hsp70s: PfHsp70-3 (cyan) and human Grp75 (magenta) exhibit high structural conservation in their SBDs. **e** Canonical, parasite ER Hsp70: PfHsp70-2 (cyan) shows minor structural variations from its homologue HspA5 (purple). **f** Exported parasite protein, PfHsp70-x (cyan) share high structural similarity with human HspA1A (magenta)

In general, structural analysis of the SBDs of human and *P. falciparum* Hsp70 homologues showed greater variation within the non-canonical Hsp70s as compared to their canonical counterparts (Fig. 6). PfHsp70-y and its human homologue, Grp170, were shown to possess less conserved SBDs. Grp170 and Hsp110 are also known to exhibit unique substrate preferences compared to canonical Hsp70s (Xu et al. 2012). The substrate-binding residues flanked by the SBD loops L_1_,_2_ and L_3,4_ are thought to be responsible for imparting substrate specificity (Xu et al. 2012). The same residues are conserved in loops L_1_,_2_ and L_3,4_ of the canonical Hsp70s. Variations in these could account for varied substrate preferences between canonical and non-canonical Hsp70 types.

It was also interesting to note that in all the Hsp70s, most variations occur in the loop regions of the substrate-binding cleft compared to the helical lid sections. This may suggest that the loops may influence functional specificity of Hsp70 as has been reported recently (Mabate et al. 2018). Furthermore, human Hsp110 (HspH1) and PfHsp70-z exhibited minor variations in the SBD. PfHsp70-z exhibits slightly longer loops located in the substrate-binding cleft as compared to human HspH1 (Fig. 6). While canonical Hsp70s generally show high conservation, human Grp170 exhibits a β-sheet protrusion located within its SBDβ which is fairly distinct from that of its counterpart, PfHsp70-y (Fig. 6). Human Grp75/HspA9 also possesses a helical fold around β5 and β6 segments of the SBC, while PfHsp70-3 has a loop in the same region (Fig. 6). These variations could possibly dictate substrate binding preferences. Notably, the SBD of PfHsp70-2 showed some degree of structural divergence within the SBC region as it seemed to possess shorter loops than its human homologue, HspA2. This could suggest that these ER homologues may possess specialized functions representing possible unique protein folding requirements between parasite and human systems.

## Signature motifs of *P. falciparum* Hsp70s and their human homologues

Despite possessing a highly conserved domain architecture, plasmodial Hsp70s are thought to be tailored for species-specific functional demands (Pryzborski et al. 2015). Outlined below are the unique structural features that delineate *P. falciparum* Hsp70s and their human counterparts.

### *P. falciparum* Hsp70s possess a valine residue preceding the EEVD motif

**Fig. 7:**
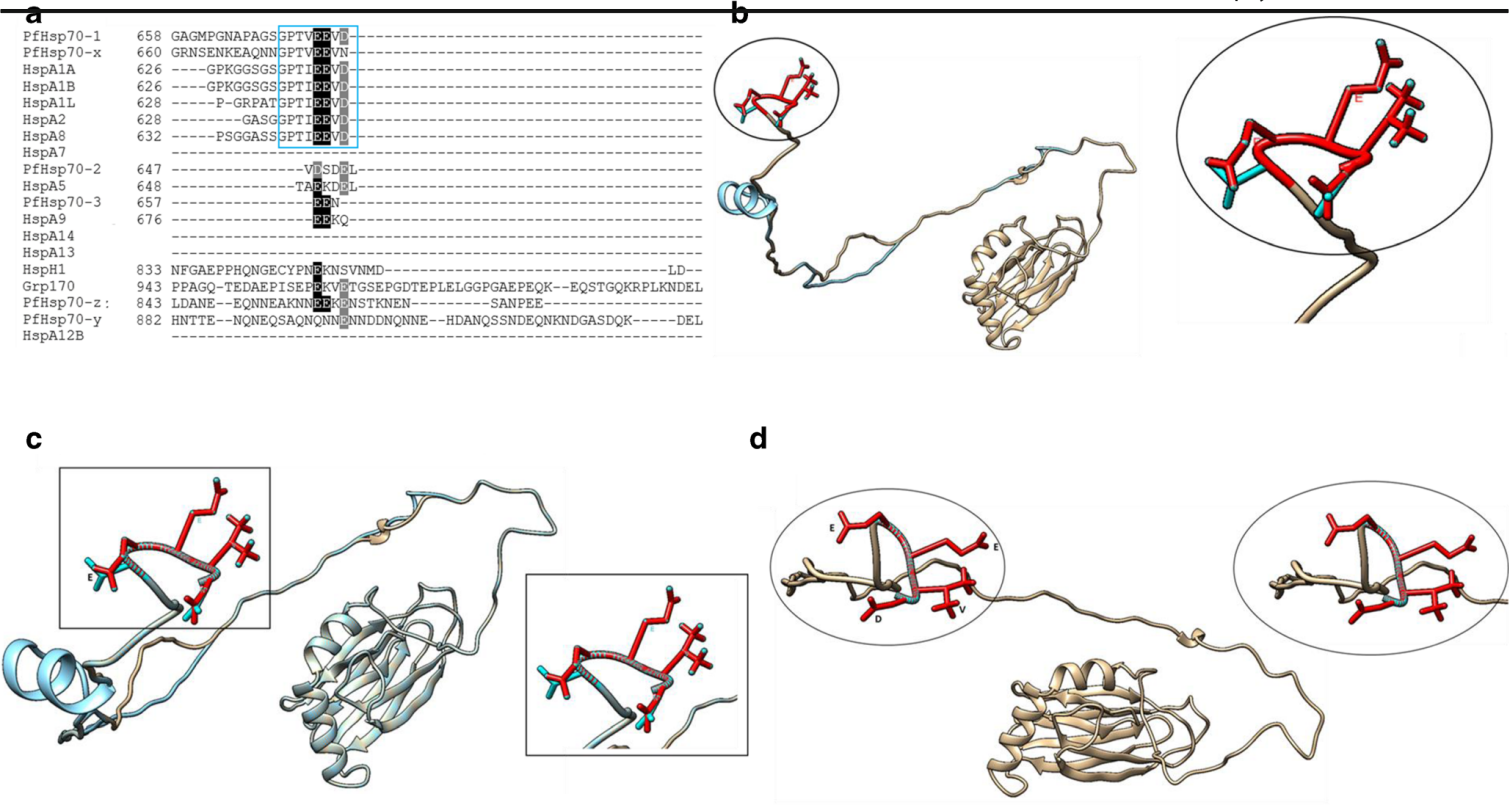
The Hsp70 EEVD/N motif. The C-terminal residues EEVD/EEVN of plasmodial and human Hsp70s PfHsp70-1 and PfHsp70-x possess a valine residue before the EEVD motif, while all human proteins possess isoleucine residues (**a**). The EEVN (cyan) and EEVD (red) motifs of PfHsp70-x and PfHsp70-1, respectively, show structural conservation (**b**). The EEVN (cyan) motif and the EEVD motif of Hsc70 show structural conservation (**c**). Human EEVD motifs exhibit high conservation amongst themselves (HsA1A-red; Hsc70-cyan) (**d**)

Human and *P. falciparum* cytosol-localized canonical Hsp70s possess the negatively charged EEVD motif to which Hop, the module that brings Hsp70 and Hsp90 into a functional complex, anchors via its tetratricopeptide repeat (TPR) domains (Zininga et al. 2015). However, unlike their human counterparts, PfHsp70-1 and PfHsp70-x possess a valine instead of an isoleucine residue which precedes the C-terminal EEVD/N motif (Fig. 7). The C-terminal TVEEVD motif of Ssa1 has recently been reported to function as a SUMO-interacting motif (Gong et al. 2018). This suggests that PfHsp70-1 could potentially be involved in SUMOylation. SUMOylation is essential for normal cell function and a potential target of small molecule inhibitors against *P. falciparum* (Reiter and Matunis 2016).

### GGMP-repeat motif

**Fig. 8:**
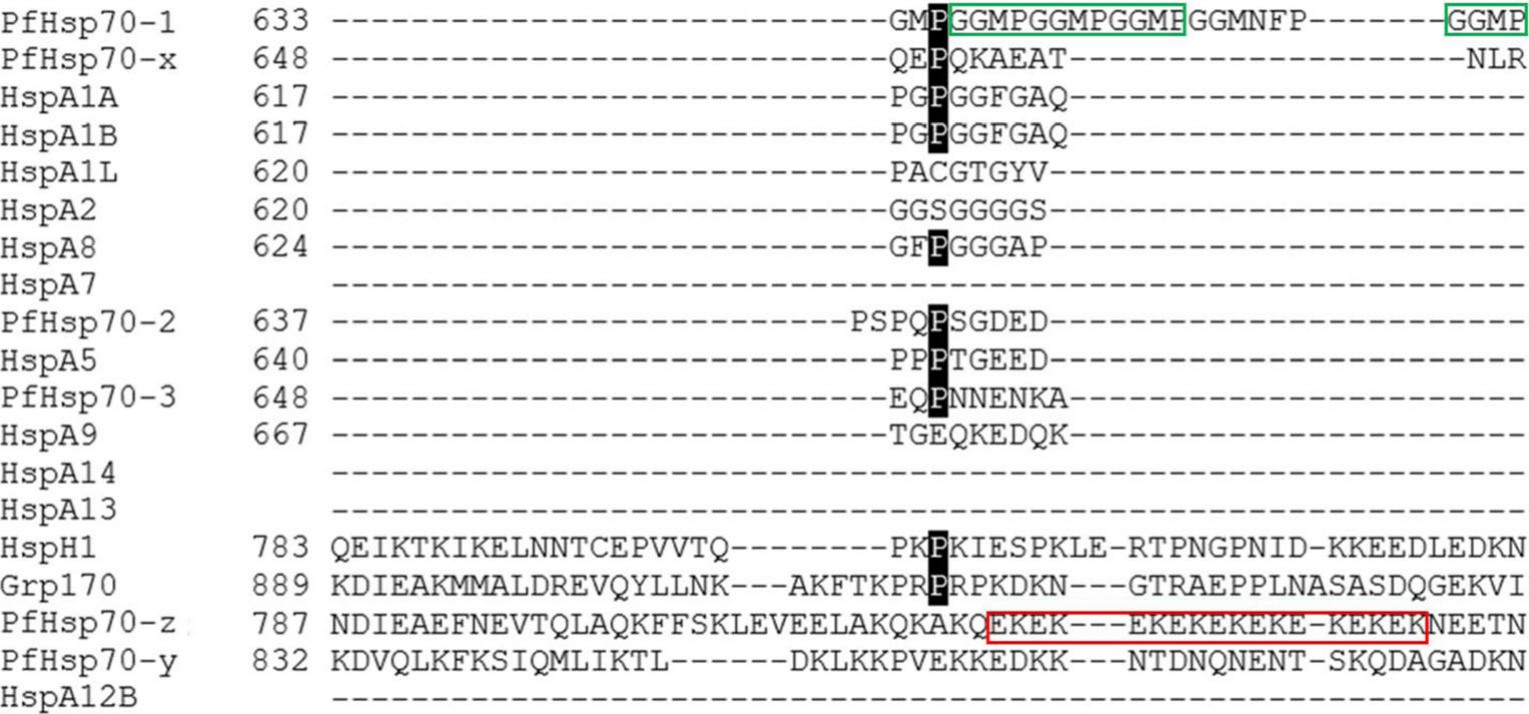
The GGMP and EKEK repeat motifs on the c-termini of PfHsp70-1 and PfHsp70-z. The C-terminal end of Hsp70s is characterized by sequence divergence and the GGMP repeat motif of PfHsp70-1 is highlighted in green while the EKEK repeat motif of PfHsp70-z is highlighted in red

Of all the 6 *P. falciparum* Hsp70 isoforms, PfHsp70-1 is noted for possession of unique GGMP repeat motifs positioned towards its C-terminal in the lid segment. This motif is only present in PfHsp70-1 and absent from other *P. falciparum* Hsp70 isoforms. In addition, it is absent in human Hsp70 homologues except in the Hsc70 homologue (P11142) which possesses a short ^619^GGMPGGMP^626^ motif, representing only two GGMP repeats compared to five GGMP repeats and an additional GGMN segment in PfHsp70-1 (Fig. 8). Thus, the enhanced presence of GGMP repeats in PfHsp70-1 is a distinct feature of this essential protein (Chiang et al. 2009). A closely related motif, the GGAP repeat present in yeast Hsp70 (Ssa1), was recently reported to act as a secondary peptide binding site, hence is thought to regulate the substrate binding specificity of Ssa1 (Gong et al. 2018). In addition, the GGAP motif of Ssa1 was implicated in Hsp40 co-chaperone binding (Gong et al. 2018). Hence, it is possible that the GGMP repeat motifs of PfHsp70-1 could similarly regulate both substrate recognition and co-chaperone binding. In *Toxoplasma gondii*, the GGMP repeat motif of Hsp70 was reported to be associated with parasite virulence (Lyons and Johnson 1998). Ssa1 ΔGGAP and GGAG mutants were shown to exhibit reduced thermo-tolerance, thus demonstrating the importance of this motif in Hsp70 function (Gong et al. 2018).

### EKEK-repeat motif of PfHsp70-z

We also identified here a novel 18mer EKEK repeat motif on the C-terminus of PfHsp70-z (Fig. 8). This feature is located between positions ^810^EKEK--K^827^ of PfHsp70-z and is one of the features that distinguishes this protein from other Hsp70 homologues (Fig. 8). This highly charged region could potentially play a role in facilitating electrostatic interactions with functional partners. It may also influence the stability of the molecule, since C-terminal segments of Hsp70 have previously been reported to confer stability to the protein (Misra and Ramachandran 2009; Mabate et al. 2018).

### TEDWYLEE and Magic motifs

**Fig. 9:**
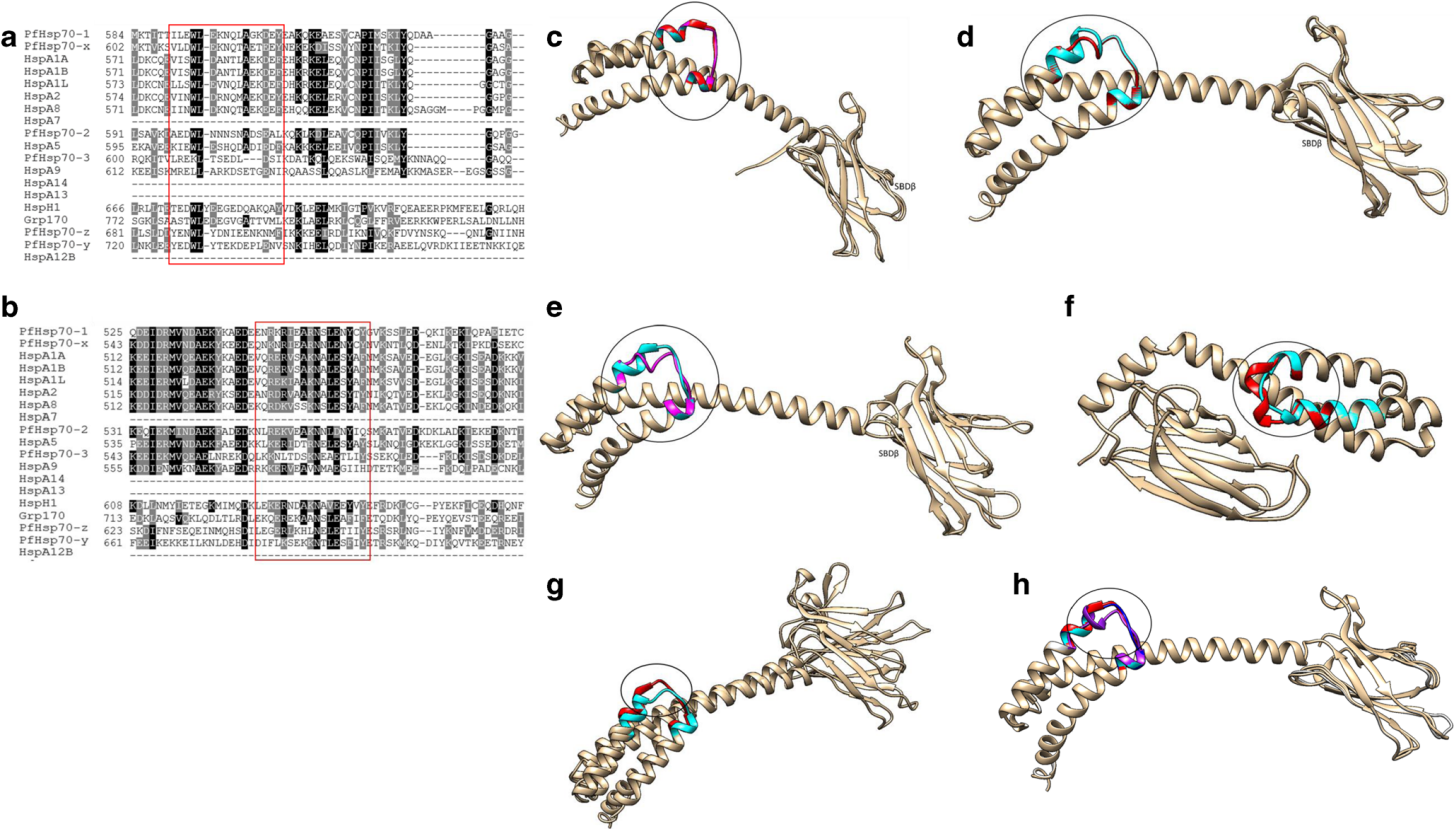
Tedwlyee and Magic motifs in *P. falciparum* and human Hsp70s. The Hsp70 Tedwlyee motifs of plasmodial and human Hsp70s exhibit high sequence variation (**a**). Magic motifs are highly divergent (**b**). Both motifs are absent in HspA7, HspA14, HspA13 and HspA12B. Superimposed SBDs of Tedwlyee motifs**.** Cytosolic Hsp70s; PfHsp70-1 (red), Hsc70 (cyan) and HspA1A (magenta) (**c**), Tedwlyee motifs of PfHsp70-z (red) and human Hsp110 (blue) (**d**). PfHsp70-1 (cyan) and PfHsp70-z (magenta) (**e**), PfHsp70-3 (cyan) and HspA9 (red) **f**. PfHsp70-2 (red) and human BiP and **h**. PfHsp70-x (purple), PfHsp70-1 (red), Hsc70 (cyan) and HspA1A all show structural variation (**g**)

Human and *P. falciparum* Hsp70s both possess Magic and Tedwlyee motifs which are thought to be involved in substrate or co-chaperone recognition (Easton et al. 2000; Fig. 9). The Magic motifs are more conserved as compared to the TEDWYLEE motifs across the various Hsp70 isoforms (Fig. 9). While cytosolic Hsp70s generally exhibit high structural conservation, the TEDWLYEE motifs of Hsp110s and ER Hsp70s assume distinct structural orientations (Fig. 9). Notably, in spite of PfHsp70-3 and its mitochondrial counterpart, human HspA9 sharing highly conserved SBDs, there is variation within the TEDWLYEE motifs of these proteins. In addition, PfHsp70-z possesses a helical fold within its TEDWLYEE motif that is lacking in its canonical, cytosolic counterpart, PfHsp70-1 (Fig. 9). Compared to human and parasite cytosol localized Hsp70s, PfHsp70-x also harbours a distinct TEDWYLEE motif which is predicted to form a loop around residue, ^609^L, and a helical fold around residues ^613^EK^614^ (Fig. 9). Variation within the TEDWLYEE and Magic motifs of Hsp70 could further account for substrate specificity and may regulate interaction with co-chaperones.

## Conclusion

Although plasmodial Hsp70s generally share high sequence and predicted three-dimensional conservation with their human counterparts, there exists some unique features present in some plasmodial Hsp70s. For instance, Tedwlyee, Magic, GGMP and EKEK motifs appear to be amongst the distinctive features setting parasite Hsp70s apart from their human counterparts. Whether these distinctive features would impart sufficient structural variation to allow for selective inhibition of parasite Hsp70 remains to be fully explored. However, a study by Cockburn et al. (2014) identified small molecule inhibitors that seem to selectively target Hsp70 of parasite origin with minimum adverse effects on human Hsp70 function (Cockburn et al. 2014). It is therefore important to conduct experimental studies to validate the roles of the unique motifs described here towards validating their roles and to further explore how they regulate Hsp70 function in malaria parasite versus human systems.
